# Brief intervention to prevent fetal alcohol spectrum disorders: Russian physicians’ skills demonstrated in an educational and a clinical trial in Russia

**DOI:** 10.1186/1940-0640-7-S1-A71

**Published:** 2012-10-09

**Authors:** Tatiana Balachova, Barbara Bonner, Vladimir Shapkaitz, Galina Isurina, Larissa Tsvetkova, Irina Grandilevskaya

**Affiliations:** 1 Department of Pediatrics, University of Oklahoma Health Sciences Center, Oklahoma City, OK, USA; 2 Academy of Pediatrics, St. Petersburg Pediatric Academy, St. Petersburg, Russia; 3 Department of Psychology, St. Petersburg State University, St. Petersburg, Russia

## 

Prenatal alcohol consumption can result in a range of adverse outcomes including fetal alcohol spectrum disorders (FASDs). Russia has high alcohol use, and hazardous drinking among women has increased. Russian women reported that obstetrician-gynecologists (OB-GYNs) would be the best source of information about associated risks of alcohol use during pregnancy. Two initiatives to reduce FASD, Project CHOICES (Changing High-Risk Alcohol Use and Increasing Contraception Effectiveness Study) and the Healthy Moms intervention, as well as a brief dial-focused physician intervention (DFBPI), were modified for use in Russian OB-GYN care. We compared OB-GYN skills demonstrated in an educational trial and in a clinical trial aimed at preventing alcohol-exposed pregnancies. Sixty-five OB-GYNs participated in the educational trial through continuing medical education (CME) programs at St. Petersburg Pediatric Academy in Russia. Participants’ DFBPI skills were assessed using videotapes of roleplaying with a simulated patient. Audiotapes of 80 clinic visits conducted by 14 OB-GYNs trained in DFBPI were coded. The OB-GYNs and study participants completed exit checklists after each clinic visit. Compared with OB-GYN physicians assigned to regular CME programs, physicians who received DFBPI training had significantly improved skills and higher levels of competency in conducting alcohol screening and interventions. Discussing the difficulties and barriers that may prevent women from achieving alcohol abstinence/reduction goals appeared to be the most difficult component for physicians to implement, and OB-GYNs had difficulty assisting patients in discussing barriers and selecting contraception methods. Russian physicians trained in DFBPI were able to implement skills learned in the clinical trial. In addition to the alcohol focus, a component to improve physicians’ skills in discussing contraceptive use should be added to DFBPI training.

